# Follow-Up Study of Adolescents Exposed to Di(2-Ethylhexyl) Phthalate (DEHP) as Neonates on Extracorporeal Membrane Oxygenation (ECMO) Support

**DOI:** 10.1289/ehp.6901

**Published:** 2004-04-07

**Authors:** Khodayar Rais-Bahrami, Susan Nunez, Mary E. Revenis, Naomi L.C. Luban, Billie L. Short

**Affiliations:** ^1^Departments of Neonatology,; ^2^Endocrinology, and; ^3^Transfusion Medicine, Children’s National Medical Center and The George Washington University School of Medicine, Washington, DC, USA

**Keywords:** DEHP, ECMO, toxicity

## Abstract

Di(2-ethylhexyl) phthalate (DEHP) is used to make polyvinyl chloride (PVC) plastic tubing soft and flexible. Animal data show that adverse effects of DEHP exposure may include reduced fertility, reduced sperm production in males, and ovarian dysfunction in females. Known treatments that involve high DEHP exposures are blood exchange transfusions, extracorporeal membrane oxygenation (ECMO), and cardiovascular surgery. Although potential exposure to DEHP in ECMO patients is significant, the exposure has not been associated with short-term toxicity. To evaluate long-term toxicity, we undertook a study of neonatal ECMO survivors to assess their onset of puberty and sexual maturity. We evaluated 13 male and 6 female subjects at 14–16 years of age who had undergone ECMO as neonates. All subjects had a complete physical examination including measurements for height, weight, head circumference, and pubertal assessment by Tanner staging. The testicular volume and the phallic length were measured in male participants. Laboratory tests included thyroid, liver, and renal function as well as measurements of luteinizing hormone, follicle-stimulating hormone, testosterone for males, and estradiol for females. Except for one patient with Marfan syndrome, the rest had normal growth percentile for age and sex. All had normal values for thyroid, liver, and renal functions. Sexual hormones were appropriate for the stage of pubertal maturity. Our results indicate that adolescents exposed to significant quantities of DEHP as neonates showed no significant adverse effects on their physical growth and pubertal maturity. Thyroid, liver, renal, and male and female gonadal functions tested were within normal range for age and sex distribution.

Human exposure to di(2-ethylhexyl) phthalate (DEHP) occurs throughout life. Of particular concern is the exposure of fetuses, preterm infants, and babies because the developing human reproductive system may be affected when the metabolic pathways of detoxification are immature. DEHP has been shown to damage the male and female reproductive systems in newborn animals. Animal studies have shown DEHP to be particularly harmful to developing fetuses: Adverse effects in the reproductive system include changes in the testes, specifically the Sertoli cell, leading to reduced fertility and changes in sperm production in males (Foster et al. 2001; Park et al. 2002; Poon et al. 1997) and ovarian dysfunction and decreased hormone production in females (Davis et al. 1994; Lovekamp-Swan and Davis 2003). Respiratory distress and changes in kidney and liver function have also been linked to DEHP exposure (Crocker et al. 1988; Kevy and Jacobson 1982; Latini 2000; Rock et al. 1987; Roth et al. 1988; Ward et al. 1998).

DEHP derives from a family of chemicals called phthalates. These chemicals are used to make polyvinyl chloride (PVC) plastic tubing soft and flexible. Because DEHP does not bind to the plastic, it can leach out of the PVC products. DEHP is widely used in PVC disposable medical devices. As in other products, DEHP can leach out of flexible PVC medical devices into the solution or medication it contains and subsequently into the patient (Rubin and Schiffer 1976).

Species differences in toxicity and metabolism of DEHP have created considerable debate about the relevance of studies in rodents to human health. However, exposures in neonatal intensive care units (NICUs) are potentially at or above levels known to cause adverse health effects in relevant animal studies (e.g., Tickner et al. 2001). For infants requiring intensive care, DEHP exposure can occur at three orders of magnitude greater than average adult exposures and at or above levels shown to cause adverse reproductive effects in animals (e.g., Tickner et al. 2001).

DEHP concentrations in blood and blood products are of particular concern for neonates who receive regular blood transfusions. The most commonly used blood products—packed red blood cells and plasma—are typically stored in DEHP plasticized bags and administered to patients through DEHP plasticized intravenous tubes. Less common treatments that involve potentially high DEHP exposures are blood exchange transfusions and extra-corporeal membrane oxygenation (ECMO). Although potential exposure to DEHP in ECMO patients is significant, it has not been associated with short-term toxicity. To evaluate long-term toxicity, we undertook a study of adolescents who had previously undergone ECMO treatment in the neonatal period to assess their onset of puberty and sexual maturation in comparison to an age- and sex-matched reference population.

## Methods

This prospective study was approved by the institutional review board at Children’s National Medical Center. After obtaining informed consent and assent, we evaluated 19 (13 male and 6 female) adolescents 14–16 years of age who had undergone ECMO as neonates. All subjects had a complete physical examination including measurements for height, weight, head circumference, and pubertal staging according to the method of Tanner (Morris and Udry 1980; Tanner 1975). In addition, the testicular volume and the phallic length were measured in all male participants. Laboratory tests included measurements of thyroid function [thyroid-stimulating hormone, free thyroxine (T_4_) by dialysis, and T_4_], liver function (aspartate aminotransferase, alanine aminotransferase, γ-glutamyl transpeptidase, and total and direct bilirubin), renal function (blood urea nitrogen and creatinine), as well as measurements of luteinizing hormone (LH), follicle-stimulating hormone (FSH), testosterone for males, and estradiol for females.

## Results

Except for one female participant with a diagnosis of Marfan syndrome, the rest had normal growth percentiles for age and sex. All the participants had normal laboratory values for thyroid, liver, and renal functions. The levels of LH, FSH, testosterone in males, and estradiol in females were normal and appropriate for the degree of pubertal development. Results of the sex hormones related to pubertal maturation are shown in Tables 1 and 2 as mean values (normal ranges).

## Discussion

Our study did not show long-term adverse outcome related to physical growth and pubertal development in adolescents previously exposed to DEHP in the neonatal period. This is in contrast to the animal data in multiple species, which show a variety of reproductive and developmental toxicities when this plasticizer is administered both orally and parenterally.

Individuals who have among the highest exposures to DEHP are those undergoing medical treatments or procedures such as dialysis, exchange transfusion, ECMO, and cardiovascular surgery. Shneider et al. (1989) have shown that babies undergoing ECMO, in which the blood is circulating through PVC tubing, are exposed to 42–140 mg DEHP/kg body weight over a treatment period of 3–10 days (Shneider et al. 1989). Karle et al. (1997) reported a lower level of exposure that ranged from nondetectable to 34.9 mg/kg/treatment period. The nondetectable level resulted from the use of a heparin coating on the DEHP-plasticized PVC circuit. In addition to the heparin coated tubing, Karle et al. (1997) attributed the difference between their study and that of Shneider et al. (1989) to the smaller surface area of the newer ECMO configurations and the varying percentage of DEHP composition in each type of tubing.

Although intravenous exposure to DEHP through the ECMO circuit or other intravenous routes exceeds recommended oral exposure limits, it is difficult to directly compare the two because one is an assumed lifetime daily oral exposure and the other an acute temporary exposure during ECMO therapy. Also, the routes of exposure differ: oral versus intravenous (Doull et al. 1999). Because the human exposure can be similar to the doses that are toxic in rodents, there is an ongoing concern that exposure to DEHP in neonatal intensive care units may adversely affect the developing reproductive organs in these infants (Huber et al. 1996). The most sensitive system appears to be the immature male reproductive tract, especially the Sertoli cell (Parks et al. 2000; Poon et al. 1997).

When DEHP enters the human body, the compound is metabolized into various substances that are more rapidly excreted. The most important of these metabolites, monoethylhexyl phthalate (MEHP) is thought to be responsible for much of DEHP’s toxicity. The enzymes that break down DEHP into MEHP are found mainly in the intestines but also occur in the liver, kidney, lungs, pancreas, and plasma. Because conversion of DEHP to MEHP occurs primarily in the intestinal tract, exposure to DEHP by ingestion may be more hazardous than by intravenous exposure, which largely bypasses the intestinal tract (Huber et al. 1996; Lewandowski et al. 1980; Thomas et al. 1979).

Our study of adolescents exposed to significant quantities of DEHP as neonates showed no significant adverse effects of DEHP on their physical growth and pubertal maturity. Thyroid, liver, renal, and male and female gonadal functions tested were within normal range for age and sex distribution. We hypothesize that the acute and short-term exposure to DEHP in an intravenous form and lack of significant conversion of DEHP to MEHP may be protective against its long-term side effects.

## Figures and Tables

**Table 1:** Results of sexual hormones in female subjects matched for Tanner stage [mean value (normal reference range)].

Females (*n*)	Tanner stage	LH (IU/L)	FSH (IU/L)	Estradiol (pg/mL)
4	4	6.05 (0.72–15.01)	4.58 (1.26–7.37)	48.75 (25–345)
2	5	3.7 (0.30–29.38)	2.65 (1.02–9.24)	118.5 (25–410)

**Table 2:** Results of sexual hormones, testicular volume, and phallic length in male subjects matched for Tanner stage [mean value (normal reference range)].

Males (*n*)	Tanner stage	LH (IU/L)	FSH (IU/L)	Testosterone (ng/dL)	Testicular volume (mL)	Phallic length (cm)
4	2–3	1.83 (0.26–3.74)	2.40 (0.72–10.37)	119 (15–280)	11 (5–10)	8.0 (6.3–8.6)
9	4–5	3.02 (0.55–7.00)	3.61 (1.70–7.00)	387 (105–800)	22 (20–29)	11.2 (8.6–9.9)
